# Feasibility and Acceptability of Digital Cognitive Screening Approaches for Older Adults in Primary Care

**DOI:** 10.1002/alz.092820

**Published:** 2025-01-09

**Authors:** Louisa I. Thompson, Molly Lawrence, Jennifer A Rosenbaum, Stephanie Czech, David C Anthony, Rabin F Chandran, Arnold R Goldberg, Andrey Vyshedskiy, Anashua R Elwy, Charles B Eaton

**Affiliations:** ^1^ Butler Hospital Memory and Aging Program, Providence, RI USA; ^2^ Alpert Medical School of Brown University, Providence, RI USA; ^3^ Boston University, Boston, MA USA

## Abstract

**Background:**

New immunotherapies for early‐stage Alzheimer’s disease (AD) have ushered in fresh hope for AD research and clinical care, but also highlight barriers to AD screening and timely diagnosis in the US. Digital cognitive assessments could potentially streamline screening and referrals for AD treatment and/or clinical trials. We report preliminary data on the feasibility and acceptability of three digital cognitive approaches for older adults completing Annual Wellness or routine follow‐up visits with a primary care provider (PCP).

**Methods:**

Data were collected for an ongoing primary care‐based study in Rhode Island. Cognitive screening approaches included: 1) remote online screening with the Boston Online Cognitive Assessment (BOCA) prior to the PCP appointment, 2) self‐administered screening with the BOCA in the waiting room immediately before or after the appointment, and 3) provider‐administered screening during the appointment using the Digital Clock and Recall (Linus Health DCR^TM^). Participants also completed a 30‐minute, in‐clinic cognitive health consultation including the Montreal Cognitive Assessment (as a reference standard) to receive feedback and referral options. Five PCPs aided in protocol development through focus groups and participated in data collection. Potential participants were identified via EMR. Recruitment included mailings, MyChart messages and phone calls, and direct referrals from PCPs. Exclusion criteria: dementia or other neurological disease, score of <13 on the telephone version of the MoCA.

**Results:**

34 older adults were screened, 23 enrolled, and 2 withdrew. The sample is currently 52% female, 81% White, and mean age is 66. Most participants completed remote BOCA screening on a smartphone. Most reported that they preferred screening at home compared to in the clinic. 11 out of 11 participants completed at least one instance of the BOCA screening online prior to their PCP appointment. DCR tablet screening was successful in 9 out of 11 PCP appointments. PCP time constraints and technological issues were reasons for incomplete administration. In‐clinic self‐administered BOCA screening was discontinued due to space and time constraints, and the lack of a designated coordinator onsite.

**Conclusions:**

Both remote online screening and tablet‐based, provider‐administered screening in‐clinic may be feasible and acceptable approaches to cognitive screening for older adults in primary care.

